# Antenatally detected urinary tract dilatation: a 12–15-year follow-up

**DOI:** 10.1007/s00467-020-04659-4

**Published:** 2020-06-23

**Authors:** Maria Herthelius, Rimma Axelsson, Karl-Johan Lidefelt

**Affiliations:** 1 Astrid Lindgren Children’s Hospital, Karolinska University Hospital, Stockholm, Sweden; 2 Department of Clinical Science, Intervention, and Technology, Division of Pediatrics, Karolinska Institutet, Stockholm, Sweden; 3 Function and Imaging, Medical Physics, and Nuclear Medicine, Karolinska University Hospital, Stockholm, Sweden; 4 Department of Clinical Science, Intervention, and Technology, Division of Radiology, Karolinska Institutet, Stockholm, Sweden

**Keywords:** Hydronephrosis, Urinary tract dilatation, Follow-up, DMSA, Children

## Abstract

**Background:**

Antenatally diagnosed urinary tract dilatation (UTD) still burdens healthcare providers and parents. This study was conducted to establish long-term outcome in an unselected group of children with antenatally detected UTD.

**Methods:**

Seventy-one out of 103 children born in 2003–2005 and diagnosed with antenatal UTD agreed to participate in a 12–15-year follow-up including blood and urine samples, a kidney ultrasound exam, and kidney scintigraphy. The records were searched for previous urinary tract infections.

**Results:**

Among children with an anteroposterior diameter (APD) ≤ 7 mm and no calyceal, kidney, ureteral, or bladder pathology in the early postnatal period, no one tested had reduced estimated glomerular filtration rate (eGFR), albuminuria, or UTD at the follow-up at a mean age of 13.6 years. One child had kidney damage not affecting kidney function. Among children with postnatal APD > 7 mm and/or kidney, calyceal, ureteral, or bladder pathology, 15% had persistent UTD and 32–39% (depending on the method used) had kidney damage. Major postnatal urinary tract ultrasound abnormalities and a congenital anomalies of the kidney and urinary tract (CAKUT) diagnosis were factors associated with an increased risk for permanent kidney damage (odds ratios 8.9, *p* = 0.016; and 14.0, *p* = 0.002, respectively). No one had reduced eGFR. One child (1/71, 1%) had a febrile urinary tract infection after the age of 2.

**Conclusions:**

We conclude that in children with postnatal APD ≤ 7 mm, no calyceal dilatation, normal bladder, ureters, and kidney parenchyma, the outcome is excellent. There is no need for long-term follow-up in these patients.

## Introduction

The question of what is the optimal postnatal follow-up for antenatally detected urinary tract dilatation (UTD) in children is still being debated [1–3]. Early postnatal investigations usually identify children with urological abnormalities in need of follow-up during childhood. The prognosis and long-term outcomes for this group of children are well documented [4, 5]. In the majority of children with antenatally detected UTD, no underlying pathology will be identified postnatally [6]. In these children, UTD is regarded as a transient and benign condition [4, 6], but uncertainty regarding the cause and prognosis may nevertheless lead to concern among the parents.

Only few studies have followed children with antenatally detected UTD for longer than a median of 2 years [5, 7, 8], and even fewer for longer than a median of 10 years [9, 10]. In these studies, the included cases had a wide range of follow-up times [5, 7–10], and some of them only examined subgroups of children [7, 8, 10]. To meet the need for a study of an unselected cohort with a long follow-up [11], we conducted this 12–15-year re-evaluation of children antenatally diagnosed with UTD, born in 2003–2005, and followed according to a standardized protocol during the first 2 years of life [12–14].

## Patients and methods

The present study is a follow-up of a population-based cohort study of children with antenatally detected UTD, born in the catchment area of Karolinska University Hospital, Stockholm, Sweden, between 2003 and 2005. The patients and methods have been described elsewhere [12–14].

Kidney pelvis anteroposterior diameter (APD) was measured at routine ultrasound screening at gestational week 19 and, if dilated, again at gestational week 32. In 14,000 consecutive pregnancies, 106 fetuses (0.7%) were diagnosed with UTD, defined as an APD of ≥ 5 mm at any time during the pregnancy. Postnatally, 103 children were followed according to a standardized protocol including ultrasound at 5–7 days, 3 weeks, and 3, 6, and 12 months; a voiding cystourethrogram (VCUG) at 6–8 weeks; and a dimercaptosuccinic acid (DMSA) scintigraphy at 2 years of age.

The cohort was divided into two groups according to the findings of the first two postnatal ultrasound investigations. Children with a postnatal APD of ≤ 7 mm, normal kidney parenchyma, and no ureteral and/or calyceal dilatation were assigned to group A (subgroup A in this article), and children with a postnatal APD > 7 mm and/or other abnormalities on the first two ultrasound investigations were designated as group B (subgroup B). Results from the 2-year follow-up have been published elsewhere [12–14].

The 12–15-year follow-up study protocol included blood and urine tests, a kidney ultrasound, and a DMSA scan. Estimated glomerular filtration rate (eGFR) was calculated from plasma creatinine according to the updated bedside Schwartz formula [15]. Urine was analyzed for blood, albumin, leukocytes, and glucose. Albuminuria was measured using the urine albumin creatinine ratio and was regarded as significant at ratios > 50 mg/mmol. All tests and investigations were optional, and the children were free to decline participation in any part of the study.

Parents and children were asked about previous urinary tract infections (UTIs), febrile and non-febrile, and the medical records were searched retrospectively for additional information. Urinary tract infection was defined as significant bacteriuria (≥ 10^5^ colony-forming units/mL) combined with symptoms consistent with UTI, for which antibacterial therapy was prescribed. Episodes of UTI associated with fever ≥ 38.5 °C were referred to as “febrile UTI” and all others as “non-febrile UTI.”

At the 1-year follow-up, UTD was defined as an APD of ≥ 7 mm, and at the 12–15-year follow-up as an APD of ≥ 10 mm.

Parenchymal abnormalities were defined as abnormal parenchymal thickness or appearance on ultrasound examination, as judged by the examiner.

“Major postnatal urinary tract ultrasound abnormalities” were defined as any of the following findings, alone or in combination: APD ≥ 15 mm, peripheral calyceal dilatation, abnormal parenchymal thickness or appearance, abnormal ureters, and abnormal bladder at the first two postnatal ultrasounds [2].

Permanent kidney injury was defined as a focal uptake defect in one or both kidneys or a split kidney function of < 45% in one kidney on the DMSA scan, according to the definitions by Piepsz et al. [16], or a single kidney status due to unilateral aplasia, dysplasia, or previous nephrectomy or other parenchymal abnormalities according to ultrasound examination.

Continuous variables with a normal distribution are expressed as mean ± standard deviation (SD), and categorical data are expressed as proportions. Intergroup comparisons of means were analyzed using Student’s *t* test and comparisons of proportions using Fisher’s two-tailed test. Associations between predictors and outcome were assessed using logistic regression. *P* values < 0.05 were considered significant.

This study was approved by the regional ethics committee and by Karolinska University Hospital’s Radiation Protection Committee.

## Results

**Fig. 1:**
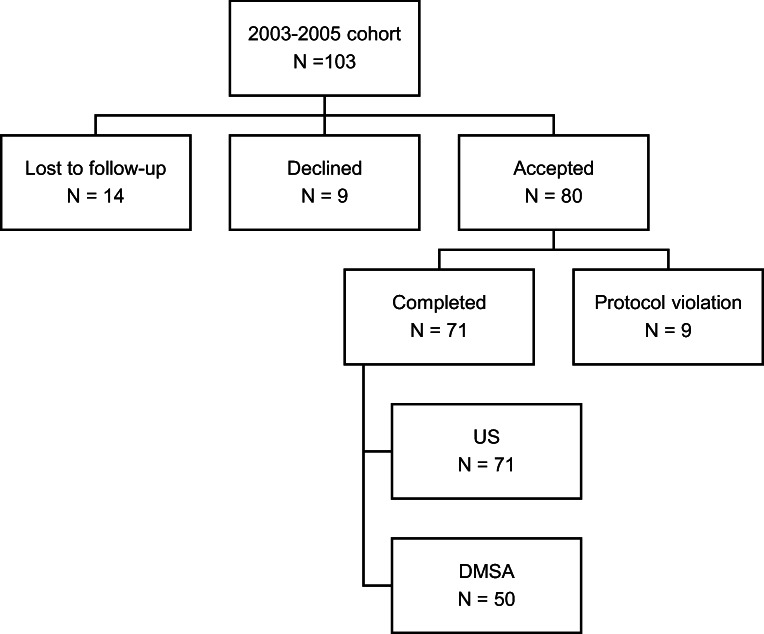
Flowchart of 103 eligible children. DMSA, dimercaptosuccinic acid scan; N, number; US, ultrasound examination

A flowchart of the 103 eligible patients is presented in Fig. 1. Eighty children were included in the study. Nine did not comply with the protocol and were subsequently excluded, which left 71 children for analysis. Fifty-two were boys (52/71, 73%) and 19 were girls. Mean age was 13.6 ± 0.7 years. Thirty-one of the included children (31/71, 44%) were in subgroup A, and 40 (40/71, 56%) were in subgroup B.

No one in subgroup A had a diagnosis of congenital anomalies of the kidney and urinary tract (CAKUT), except one child with vesicoureteral reflux (VUR) grade I. Altogether, 15 out of 40 patients (38%) in subgroup B had a CAKUT diagnosis, three with unilateral aplasia or dysplasia, two with dysplasia and non-dilated VUR, four with VUR grades IV–V, five with ureteropelvic junction obstruction, and one with megaureter. Six children (6/40, 15%) in subgroup B required surgery during early childhood: unilateral nephrectomy in two, pyeloplasty in one, bilateral Cohen reimplantation in two, and bilateral Deflux injection for VUR grade V in one. No child in subgroup A needed surgery for urological issues.

Ultrasound and DMSA findings, the frequency of late febrile UTI, and kidney damage and function were compared between subgroup A (*n* = 31) and subgroup B (*n* = 40) (Table 1). Mean age and sex distribution did not differ significantly between the groups.

**Table 1:** Characteristics and outcome in subgroups A and B at the 12–15-year follow-up

	Subgroup A	Subgroup B
Patients	31	40
Boys (%)	21/31 (68%)	31/40 (78%)
Mean age, years	13.5	13.6
eGFR < 90 mL/min/1.73 m^2^	0/22 (0%)	0/31 (0%)
Febrile UTI(s) after age 2	0/31 (0%)	1/40 (2%)
US examination		
APD > 10 mm	0/31 (0%)	6/40 (15%)
Parenchymal abnormalities	0/31 (0%)	5/40 (12%)
DMSA		
DMSA pathology	1/19 (5%)	12/31 (39%)

*APD*, anteroposterior diameter; *DMSA*, dimercaptosuccinic acid (scan); *eGFR*, estimated glomerular filtration rate; *US*, ultrasound; *UTI*, urinary tract infection

### Ultrasound findings

All children (71/71, 100%) agreed to undergo an ultrasound examination. Results and comparisons with the 1-year follow-up are presented in Table 2. In subgroup A, all 12–15-year examinations were, as previously, normal. In subgroup B, two children (2/40, 5%) had recurrence of UTD and four (4/40, 10%) had persistent UTD, and in six (6/40, 15%), the condition had normalized compared with the 1-year ultrasound exam. No child had an anteroposterior pelvis diameter of > 22 mm at the 12–15-year follow-up. One child with a postnatal unilateral UTD of 80 mm had been nephrectomized. Four children had kidney parenchymal abnormalities (unilateral aplasia in two and unilateral dysplasia in two). All other children in subgroup B had normal ultrasound exams at both the 1-year and the 12–15-year follow-up. In summary, UTD was found in six (15%) and kidney parenchymal abnormalities in five (12%) out of 40 children in subgroup B at the 12–15-year ultrasound follow-up.

**Table 2:** Ultrasound findings showing change over time in 71 children examined at 1 year and 12–15 years of age

Ultrasound			Subgroup A	Subgroup B	Total
			*N* = 31	*N* = 40	*N* = 71
1 year		12–15 years			
Normal	→	Normal	31	23	54
Abnormal	→	Normal	0	6	6
Abnormal	→	Abnormal	0	9	9
Normal	→	Abnormal	0	2	2

*N*, number of patients examined

Normal = normal kidney parenchyma, no calyceal dilatation or dilatation of the kidney pelvis or ureters, and normal bladder appearance. Abnormal = other findings

Arrows indicate change from one status to the other

### Dimercaptosuccinic acid scintigraphy

Fifty (50/71, 70%) children agreed to undergo a DMSA scan. Results and comparisons with the previous follow-up are presented in Table 3. Among subgroup A children examined by DMSA scan, one child with a previously normal DMSA scan was (unexpectedly) diagnosed with new, generalized kidney damage at the 12–15-year follow-up. In all other subgroup A children examined by DMSA, the result was normal. The child with kidney damage in subgroup A had had bilateral UTD with APD of 9 and 11 mm, respectively, at the antenatal third trimester ultrasound exam. Postnatal ultrasounds at 1 and 3 weeks of age had been normal, and, as the assignment to the group A or B was based on these two ultrasound exams, this child had been assigned to group A. Further follow-up had shown a small bladder diverticulum on VCUG examination, a discrete calyx dilatation at the 6-month ultrasound, and a normal 1-year ultrasound. The 2-year DMSA had been normal, with no uptake defects and a split function of 45%:55%. By contrast, at the 12–15-year follow-up, this figure was 41%:59%.

**Table 3:** Findings of the dimercaptosuccinic acid (DMSA) scans showing change over time in 50 children examined at 2 years and 12–15 years of age

DMSA			Subgroup A	Subgroup B	Total
			*N* = 19*	*N* = 31*	*N* = 50*
2 years		12–15 years			
Normal	→	Normal	18	18	36
Abnormal	→	Normal	0	1	1
Abnormal	→	Abnormal	0	8	8
Normal	→	Abnormal	1	4	5

*N*, number of patients examined

*Not all patients agreed to undergo a DMSA scan

Normal = no focal parenchymal uptake defects and a split kidney function of both kidneys within the 45–55% range. Abnormal = other findings

Arrows indicate change from one status to the other

In subgroup B, eight patients (8/31, 26%) had persistent damage and four (4/31, 13%) had newly diagnosed damage. All other patients in the subgroup B had normal or (in one case) normalized DMSA scans at the 12–15-year follow-up (compared with the 2-year follow-up).

Altogether, one out of 19 children (5%) in subgroup A and twelve out of 31 children (39%) in subgroup B had signs of kidney damage on DMSA scintigraphy at the 12–15-year follow-up.

### Permanent kidney injury

Permanent kidney injury, as assessed by DMSA exam or (if no DMSA scan was done) ultrasound, was found in 14 children altogether (14/71, 20%) and was significantly more common in subgroup B than in subgroup A (13/40, 32% vs. 1/31, 3%, *p* = 0.002).

In subgroup B, permanent kidney injury assessed by DMSA only was found in 39% (12/31) of the children compared with 32% (13/40) when assessed by either DMSA or (in cases where no DMSA was done) ultrasound. Four of the 13 subgroup B children with kidney injury at the 12–15-year follow-up (31%) showed progress of kidney parenchymal damage compared with the 2-year follow-up. One had a new focal defect on the 12–15-year DMSA scan, and the other three showed worsening of split kidney function in one of the kidneys (from 47 to 44%, from 50 to 43%, and from 45 to 36%, respectively). All four had had significant UTD (11–18 mm) in the third trimester. The DMSA scans at the 2-year follow-up were normal in all these four children.

Major postnatal urinary tract ultrasound abnormalities, a CAKUT diagnosis, and female sex were tested as possible risk markers for future permanent kidney damage. Using logistic regression, major postnatal urinary tract ultrasound abnormalities (odds ratio (OR) 8.9, 95% confidence interval (CI) 1.5–52.2, *p* = 0.016) and a CAKUT diagnosis (OR 14.0, CI 2.6–74.5, *p* = 0.002), but not female sex (OR 1.6, CI 0.2–11.6, *p* = 0.631), were identified as risk markers.

Twenty-two of the subgroup A children (22/31, 71%) and 31 of the subgroup B children (31/40, 78%) agreed to blood and urine sampling. None of these children had significant albuminuria, and all had normal eGFR at the 12–15-year follow-up.

### Febrile urinary tract infections

In subgroup A, no child was on continuous antibiotic prophylaxis and no child developed a febrile UTI between the 2-year and the 12–15-year follow-up. In subgroup B, 15 children (15/40, 37.5%) were on continuous antibiotic prophylaxis at the 2-year follow-up, but no one continued prophylaxis beyond 4 years of age. One girl in subgroup B experienced one episode of febrile UTI at the age of 12. This girl had had persistent left UTD of 20–24 mm during the first 2 years of life, but a diuretic renogram showed normal split function and no significant obstruction. At the 12–15-year follow-up (approximately 6 months after the abovementioned UTI), she had a 12-mm left-side UTD but still no signs of parenchymal damage on DMSA.

## Discussion

This study shows that, in Sweden, the prognosis for children with antenatally diagnosed UTD is generally good. In our unselected, population-based cohort, no child was identified with reduced eGFR or significant albuminuria at 12–15 years (mean 13.6 years) of follow-up. Urinary tract dilatation was present in six out of 71 children (8%), and 14 out of 71 (20%) had permanent kidney injury. Only one child (1/71, 1.4%) developed a febrile UTI after 2 years of age.

Among children with a postnatal APD of ≤ 7 mm, normal kidney parenchyma, and no ureteral or calyceal dilatation (subgroup A), all but one had normal kidneys and a normal urinary tract at the 12–15-year follow-up. This case supports previous findings that screening with two postnatal ultrasounds will identify most, but not all, children in need of postnatal follow-up, as previously stated by Matsui et al. [9], and is in line with other studies reporting a low frequency of urological abnormalities in children with mild pre- and postnatal UTD [4, 17].

In subgroup B, no UTD was found at either the 1-year or the 12–15-year follow-up in 70% of children. During the same time period, UTD improved in 15%, was stable in 10%, and worsened in 5%. The proportion of children with UTD worsening over time, 5%, is higher than that, 1%, reported by Matsui et al. [9] and can be explained by the uncertainty caused by the smaller cohort in our study compared with 344 patients in the study by Matsui et al. However, even though those who worsen over time represent a minority, they illustrate the dynamic nature of UTD and emphasize the importance of not dismissing “high-risk” children too early.

In patients with antenatally detected hydronephrosis, permanent kidney damage is either congenital or acquired, but both are of equal importance for the kidney outcome. In a meta-analysis of studies with short to moderate follow-up times, Lee et al. in 2006 [4] reported the overall risk for postnatal pathology to be 36%, which is in line with the 32–39% (depending on the method used) in subgroup B in our study. Altogether, 14 children (from both subgroups A and B and diagnosed by either DMSA or ultrasound) had signs of permanent kidney injury at the 12–15-year follow-up. In most of them (9/14, 64%), kidney injury was diagnosed already at the 1- or 2-year follow-up, but in as many as 36% (5/14), signs of kidney damage first appeared at the 12–15-year follow-up. All new kidney damage at the 12–15-year follow-up was diagnosed by DMSA. Whether this, in some cases minor, kidney injury will be significant for the child’s future health is unclear, but in a recent publication, Calderon-Margalit et al. reported that a history of any childhood kidney disease (including hydronephrosis) is associated with a four-fold increased hazard ratio for chronic kidney disease stage 5 later in life [18].

Many studies have tried to define which children with antenatally diagnosed UTD will eventually need surgery, and most authors agree that children with low-grade UTD (Society for Fetal Urology (SFU) grades 1 and 2) will probably not need surgery for UTD-related issues [4, 8–10]. In our study, no child from subgroup A and six children from subgroup B (6/40, 15%) needed UTD-related surgery, which is consistent with these studies.

A question equally important to that of the need for surgery is who will require extended follow-up because of kidney injury, and who will not. In a recent publication by Costa et al. [7], the probability of kidney injury (defined as hypertension, proteinuria, and/or reduced GFR) at 15 years of age was 0%, 15%, and 24% for low-, medium-, and high-risk groups, respectively. We used the same inclusion criteria as Costa et al. (an APD ≥ 5 mm at any time during pregnancy) but did not exclude patients with megaureter, megacystis, and dysplasia as they did. Despite this, no children in our study were identified with either proteinuria or reduced GFR. The most likely explanation for this discrepancy is the smaller number of patients included in our study compared with that of Costa et al. [7]. Other possibilities are differences in living conditions, ethnic background, prevalence of comorbidities, etc. between the two study cohorts.

The frequency of UTI in children with UTD varies from approximately 3 to 34% depending on selection criteria [5, 7, 19–25]. During the first 2 years of life, frequency of UTI was 9% in our cohort [13], which is close to the 13% frequency reported from neighboring Finland [24] and is not surprising given that the majority of our participants had mild UTD. Even though no child was on antibiotic prophylaxis beyond the age of 4, only one girl (1/71, 1%) developed a febrile UTI between the 2-year and the 12–15-year follow-up. This frequency is, as expected, lower than the 2–29% frequency reported at the early postnatal follow-up [13, 22, 24–26] and corresponds better to the frequency of UTIs reported in the normal, age-matched population [27]. Tentatively contributing to this normalization of risk within the group could be previous surgical interventions and/or physiological maturation of lower urinary tract function. Conclusions regarding this can, however, not be drawn from this study.

It is difficult to compare our results with other studies in aspects other than those mentioned above [5, 7–10, 17, 19, 28], as different definitions of pre- and postnatal UTD and different endpoints and outcomes were used. Also, most of these studies [5, 7–10, 28] are registry or cohort studies with patients included over a prolonged period but with data analyzed cross-sectionally at a certain time point and the results not differentiated based on length of follow-up.

In 2014, Nguyen et al. published a consensus document with proposals for a new grading system and postnatal follow-up for children with antenatally detected UTD [29]. The document unified different pre-existing grading systems and proposed a significant reduction in the number of follow-up investigations for most affected children. The aim was to create a common “language” to facilitate research and reduce the burden of postnatal investigations for the patients and healthcare expenditure for the society. As our previous study was conducted before this consensus document was published, we have kept our original definitions and grouping of patients in the present follow-up. If, however, our cohort were categorized according to the Nguyen et al. consensus document criteria for *antenatal presentation*, antenatal UTD measures would be categorized as UTD A1 (low risk) in 27 children (27/71, 38%) and as UTD A2 (increased risk) in the remaining 44 children (44/71, 62%). Recategorizing our cohort of children according to the consensus document criteria for *postnatal presentation* would mean that 40 children (40/71, 56%) would have been categorized as normal and would not have been further evaluated. Five children (5/71, 7%) would have been categorized as UTD P1 (low risk), 21 (21/71, 30%) as UTD P2 (intermediate risk), and five (5/71, 7%) as UTD P3 (high risk).

During the time that has elapsed since the participants in this study were first included, protocols for follow-up of prenatally detected UTD have changed considerably. As mentioned above, today, most children in our cohort would probably not have been considered for postnatal follow-up beyond, at most, two routine postnatal ultrasounds. Nevertheless, we believe that it is worth establishing long-term outcomes also in this group of children for guidance for healthcare providers, parents, and others with an interest in this field.

The major limitation of this study is the number of patients lost to follow-up (32/103, 31%). Most of these children declined to participate (*n* = 9) or did not comply with the protocol (*n* = 9). However, the caregivers of these children provided information about their general health, lower urinary tract symptoms, and UTIs. From this information and from the records, we know that no one had persisting or recurring abdominal complaints, lower urinary tract symptoms, or a history of febrile or non-febrile UTIs. The distribution of sex, age, and degree of pre- and postnatal UTD was similar among children lost to follow-up as among those who completed the study. We therefore find it unlikely that inclusion of the children lost to follow-up would significantly have altered the conclusions drawn from the present study.

A strength of this study is that it is a population-based study and that the patients were non-selected and consecutively included. Even though our cohort is relatively small, the patients in this study are likely to also represent children in other parts of Sweden.

## Conclusion

We conclude that the long-term prognosis for Swedish children with postnatal APD ≤ 7 mm, normal bladder, ureters, and kidney parenchyma is excellent and that there is no need for prophylactic antibiotics, nor for long-term follow-up. Also, most children with other postnatal findings have a favorable long-term outcome with normal glomerular filtration rate and a low incidence of febrile UTI. However, major postnatal urinary tract ultrasound abnormalities and a CAKUT diagnosis carry an increased risk for permanent kidney damage.

## Data Availability

The complete data can be obtained from the authors on request.
